# A Cross-Reactive Human Single-Chain Antibody for Detection of Major Fish Allergens, Parvalbumins, and Identification of a Major IgE-Binding Epitope

**DOI:** 10.1371/journal.pone.0142625

**Published:** 2015-11-18

**Authors:** Merima Bublin, Maria Kostadinova, Julian E. Fuchs, Daniela Ackerbauer, Adolfo H. Moraes, Fabio C. L. Almeida, Nina Lengger, Christine Hafner, Christof Ebner, Christian Radauer, Klaus R. Liedl, Ana Paula Valente, Heimo Breiteneder

**Affiliations:** 1 Department of Pathophysiology and Allergy Research, Medical University of Vienna, Vienna, Austria; 2 Institute of General, Inorganic and Theoretical Chemistry, University of Innsbruck, Innsbruck, Austria; 3 Centro Nacional de Ressonância Magnética, Instituto de Bioquímica Médica, Universidade Federal do Rio de Janeiro, Rio de Janeiro, Brazil; 4 Karl Landsteiner Institute for Dermatological Research, St. Pölten, Austria, Department of Dermatology, Karl Landsteiner University for Medical Sciences, St.Pölten, Austria; 5 Allergy Clinic Reumannplatz, Vienna, Austria; Alexion Pharmaceuticals, UNITED STATES

## Abstract

Fish allergy is associated with moderate to severe IgE-mediated reactions to the calcium binding parvalbumins present in fish muscle. Allergy to multiple fish species is caused by parvalbumin-specific cross-reactive IgE recognizing conserved epitopes. In this study, we aimed to produce cross-reactive single chain variable fragment (scFv) antibodies for the detection of parvalbumins in fish extracts and the identification of IgE epitopes. Parvalbumin-specific phage clones were isolated from the human ETH-2 phage display library by three rounds of biopanning either against cod parvalbumin or by sequential biopanning against cod (Gad m 1), carp (Cyp c 1) and rainbow trout (Onc m 1) parvalbumins. While biopanning against Gad m 1 resulted in the selection of clones specific exclusively for Gad m 1, the second approach resulted in the selection of clones cross-reacting with all three parvalbumins. Two clones, scFv-gco9 recognizing all three parvalbumins, and scFv-goo8 recognizing only Gad m 1 were expressed in the *E*. *coli* non-suppressor strain HB2151 and purified from the periplasm. scFv-gco9 showed highly selective binding to parvalbumins in processed fish products such as breaded cod sticks, fried carp and smoked trout in Western blots. In addition, the scFv-gco9-AP produced as alkaline phosphatase fusion protein, allowed a single-step detection of the parvalbumins. In competitive ELISA, scFv-gco9 was able to inhibit binding of IgE from fish allergic patients’ sera to all three β-parvalbumins by up to 80%, whereas inhibition by scFv-goo8 was up to 20%. ^1^H/^15^N HSQC NMR analysis of the rGad m 1:scFv-gco9 complex showed participation of amino acid residues conserved among these three parvalbumins explaining their cross-reactivity on a molecular level. In this study, we have demonstrated an approach for the selection of cross-reactive parvalbumin-specific antibodies that can be used for allergen detection and for mapping of conserved epitopes.

## Introduction

Fish is one of the eight most important food allergen sources which cause the majority of food-induced IgE-mediated allergic reactions [1–3]. The prevalence of fish allergy is higher in coastal countries where fish constitute a large proportion of the diet [4]. However, in the past two decades, fish consumption has undergone major changes due to the globalization of the food industry and to innovations and improvement in processing, transportation and distribution. Moreover, the consumption of fish and processed fish products has steadily increased due to the recognition of their high nutritional value [5]. The current prevalence of fish allergy ranges from 0.1% to 0.5%, but considering the increasing consumption a rise is expected [1–3, 6]. Fish allergy often persists throughout life and in allergic individuals consumption, inhalation or contact with fish and fish containing products can lead to mild local symptoms to severe systemic anaphylactic reactions [4].

IgE-mediated hypersensitivity reactions to fish are associated with β-parvalbumins, which represent the major and sole allergens for the majority of fish allergic patients [7–9]. Parvalbumins are small 12 kDa calcium-binding proteins from the EF-hand superfamily. They possess three EF-hand motifs, one non-fucntional stabilizing AB-motif, and two calcium-binding motifs, the co-called CD and EF-sites [10]. Fish-allergic patients are often sensitized to multiple fish species [8, 11, 12]. Many studies showed that this cross-reactivity was based on a predominant sensitization to epitopes on parvalbumins located on the highly conserved EF-hand motifs [9, 13–15]. However, although sequence identities between parvalbumins from the same and different fish species show a high extent of variation, [16, 17] recognition patterns of parvalbumin-specific IgE were not associated with the levels of their amino acid identities [15].

At present, the only appropriate method for patients’ treatment is avoidance of all species of fish and fish containing products. Therefore, the detection of parvalbumins in foods is of particular interest for labeling purposes and the safe-guarding of fish-allergic consumers. Furthermore, due the severity and the incurable nature of fish allergy, characterization of the IgE-binding epitopes of parvalbumins is important for understanding the molecular mechanisms underlying fish allergy and for the development of new tools for diagnosis and treatment.

The aim of this study was to produce antibodies against parvalbumins as recombinant single chain variable fragments (scFv) by phage display technology. We hypothesized that identification and selection of highly cross-reactive anti-parvalbumin antibodies could be facilitated by sequentially changing the antigen during the biopanning of the phage display library. An scFv isolated from the ETH-2 phage display library by sequential biopanning against parvalbumins from cod, carp and rainbow trout was successfully used for the detection of parvalbumins in processed fish products and for the identification of major IgE epitopes.

## Materials and Methods

### Ethics statement

The study was approved by the Ethics Committee of the State of Lower Austria. Informed written consent was obtained from all participants.

### Purification of cod, carp and trout parvalbumins

Fish filets from Atlantic cod (*Gadus morhua*), carp (*Cyprinus carpio*) and rainbow trout (*Oncorhynchus mykiss*) were purchased from a local market in Vienna, Austria. The proteins were extracted from fish tissues in 3 volumes (v/w) of destilled water and the homogenates were subsequently heated for 30 min at 60°C. After centrifugation, the supernatants were dialysed against 20 mM Tris/HCl, pH 7.5, and loaded onto a Mono Q^™^ 5/50 GL Tricon column (GE Life Science, Little Chalfont, UK). Bound proteins were eluted by a linear gradient of 0–40% 1 M NaCl in 20 mM Tris/HCl, pH 7.5, and the fractions were analysed by 15% SDS-PAGE. The identities of cod, trout and carp parvalbumins were confirmed by N-terminal amino acid sequencing performed as described by Ma et al. [18] and by immunoblotting using the mouse monoclonal anti-parvalbumin clone Parv-19 antibody (Sigma, St Louis, MO, USA) and a rabbit polyclonal anti-Gad m 1 antibody (Tepnel BioSystems Ltd, Deeside, UK).

### Preparation of extracts from raw and processed fish

Fresh carp was fried in oil (8 min, 180°C) and breaded cod sticks were heated in a microwave oven (7 min, 1 kW). Smoked trout was purchased from a local grocery store. Fresh filets of cod, carp and trout and the processed fish samples were cut and homogenized with one volume (w/v) of 10 mM phosphate buffer, pH 7.5, containing a protease inhibitor cocktail tablet (Roche, Mannheim, Germany). Proteins were extracted by stirring for 3 hours at 4°C. After centrifugation, fish extracts were analyzed by 15% SDS-PAGE and stored at -20°C.

### ETH-2 antibody phage library

The ETH-2 synthetic human antibody library contains more than 3x10^8^ clones of scFv antibodies displayed on the surface protein pIII of the filamentous phage M13 [19, 20]. It was generated by random mutagenesis of the complementary-determining regions 3 (CDR3) of only three antibody germline segments, DP-47 for the heavy chain, and DPK-22 and DPL-16 for the light chain. The diversity of the heavy chain was created by appending random loops of 4, 5 and 6 amino acid residues at position 95 of CDR3. Similarly, the diversity of the light chain was created, by randomizing six amino acid positions in the CDR3 of this chain. The ETH-2 library, which is cloned into the pDN322 phagmid vector encodes a pelB leader sequence that targets the expressed scFv to the bacterial periplasmatic space [19, 20].

### Selection of parvalbumin-binding phages by sequential antigen biopanning

Parvalbumin-specific scFvs were selected from the ETH-2 library by using the parvalbumins from cod (Gad m 1), carp (Cyp c 1) and trout (Onc m 1) as targets in three rounds of biopanning as described below (Table 1). In a control experiment three rounds of biopanning were performed only with Gad m 1.

**Table 1:** Presentation of the targets used in three rounds of sequential antigen biopanning.

Clone name	1^st^ round	2^nd^ round	3^th^ round
**scFv-gcc**	Gad m 1	Cy p c 1	Cy p c 1
**scFv-gco**	Gad m 1	Cy p c 1	Onc m 1
**scFv-goo**	Gad m 1	Onc c m 1	Onc m 1
**scFv-goc**	Gad m 1	Onc c m 1	Cyp c 1
**scFv-ggg**	Gad m 1	Gad m 1	Gad m 1

Nunc Maxisorp tubes (Nunc, Roskilde, Denmark) were coated with 1 ml of target protein (50 μg/ml) in 50 mM Na-carbonate buffer, pH 9.2 overnight, at 4°C. Coated tubes were washed with PBS and incubated with PBS containing 4% skimmed milk powder (PBSM) for 2 h at room temperature (RT). After blocking, an aliquot of the ETH-2 library, containing 2x10^12^ phages in 2 ml PBSM was added and incubated overnight. Following 10 washes with PBS containing 0.1% Tween-20 (PBST) and another 10 washes with PBS, bound phages were eluted with 1 ml of 100 mM triethylamin followed by immediate neutralization by adding 0.5 ml of 1 M Tris/HCl, pH 7.4. *E*. *coli* suppressor strain TG1 growing in mid-log phase was infected with eluted phages for titration and amplification of phages for the following rounds of selection. For amplification, phage-infected bacteria were spread on 2xTY plates containing 100 μg/ml ampicillin and 0.1% glucose (2xTY-Amp-Gluc) and incubated overnight at 30°C. After detaching the cells from the plate, 50 μl of bacterial suspension was used to inoculate 50 ml 2xTY-Amp-Gluc. Phage rescue was carried out by infecting the bacteria with 1x10^13^ M13K07 cfu of helper phage for 30 min at 37°C. The culture was centrifuged and the pellet was resuspended in 100 ml 2xTY-Amp, 50 μg/ml kanamycin. The culture was grown overnight at 30°C. Phages were precipitated with 1/5 volume 20% PEG, 2.5 M NaCl. This phage library was used for a subsequent biopanning.

Following the 3^rd^ round of biopanning, 10 individual clones were randomly chosen from each of the 3^rd^ rounds and screened for binding to cod, carp and trout parvalbumin as follows. Single ampicillin resistant *E*. *coli* TG1 colonies harbouring phagemids were inoculated in 100 ml 2xTY-Amp-Gluc, incubated for 3 h at 37°C and re-infected with 10^9^ cfu of M13K07 helper phage. After 30 min, the cultures were centrifuged and the bacterial pellets resuspended in 100 ml 2XTy-AMP, 50 mg/ml kanamycin. The following day the phages were precipitated as described above and tested by ELISA.

### Polyclonal and monoclonal phage ELISA

Phage-scFv libraries from each round of biopanning were tested for specific binding to the antigens by direct ELSA. Immunoplates were coated with 100 μl of target protein (2 μg/ml) and blocked with PBSM, and 1:200 diluted phage-scFv-libraries or 1:10 diluted individual phage clone preparations were added to the wells.

Bound parvalbumin-specific phages were detected by using a horse radish peroxidase (HRP)-labeled anti-M13 monoclonal antibody (GE Healthcare, Little Chalfont, UK). Development was performed by using SIGMA*FAST* OPD substrate tablets (Sigma-Aldrich, Steinheim, Germany). After stopping the reaction by adding of 50 μl of 0.18 M H_2_SO_4_, the absorbance was measured at 450 nm. Duplicates determinations were done for each sample.

### DNA sequencing of individual scFv clones

Single strand phagemid DNA was isolated from individual parvalbumin specific phage clones using the QIAprep Spin M13 Kit (50) (QIAGEN, Maryland, USA). For synthesis of dsDNA, *E*. *coli* XL1-Blue MRF`was transformed with the phagmid vector. After isolation of the plasmid DNA from single clones with the NucleoSpin Plasmid Kit (Macherey-Nagel, Düren, Germany), sequencing was performed using the primers fdseq1 (5´-GAA TTT TCT GTA TGA GG-3`) and DP47CDR2back (5`-TAC TAC GCA GAC TCC GTG AAG-3`) and the SequiTherm EXCEL II DNA Sequencing Kit-LC (Epicentre Biotechnologies, Madison, WI, USA). The DNA sequences were separated on a Licor 4000L sequencer and analyzed with the LICOR Image Analysis V4.0 and LICOR Align IR V2.0 software.

### Preparation of soluble scFv antibodies

*E*. *coli* non-suppressor strain HB2151 was transformed with phagemid DNA of selected clones. After induction of scFv expression by adding 1 mM isopropyl β-D-1-thiogalactopyranoside (IPTG), the bacterial culture was incubated for 18 h at 30°C. Periplasmic extracts containing scFvs were prepared by resuspending the bacterial pellet in 1/20 of the original volume of 20 mM Tris/HCl, pH 7.0, 20% sucrose and 1 mM EDTA. After incubation for 20 min on ice, the debris was removed and the supernatant dialysed against 50 mM sodium phosphate buffer, pH 8, containing 300 mM NaCl. His-tagged scFv fragments were purified on a 1 ml HisTrap HP column (GE Healthcare, Little Chalfont, UK) according to the manufacturer’s instructions. The fractions were analyzed by 15% SDS-PAGE and fractions containing scFv were pooled. After dialysis against 50 mM Na-phosphate buffer, pH 7, 50 mM NaCl, the concentration of scFv was determined using the Pierce BCA protein reagent assay.

### Expression and purification of an alkaline phosphatase-scFv fusion protein (scFv-AP)

Cloning, expression and purification of the scFv-AP were performed as described by Gruber et al. [21]. Briefly, Pst I and Not I restriction sites were incorporated at the 5´ and 3´ ends of the scFv-DNA by PCR using the pst1forward (5’-CAT CTG CAG GAG GTG CAG CTG TTG-3’) and not1rev (5’-GAT GCG GCC GCG CCT AGG ACG-3’) primers. The resulting fragment was subcloned into the expression vector pDAP2/S [22]. The resulting vector was transferred to *E*. *coli* strain XL1-Blue MRF´ and after induction of scFv-AP expression by adding 1 mM IPTG, the bacterial culture was incubated for 72 h at 18°C. After centrifugation the medium was applied to a 1 ml HisTrap HP column and purification was performed according to the manufacturer’s recommendations (GE Healthcare, Little Chalfont, UK).

### Enzyme Linked Immunosorbent Assay (ELISA)

The ability of scFv and scFv-AP to recognize parvalbumins was analysed by ELISA. Nunc maxisorp immunoplates were coated overnight with 2 μg/ml of cod, carp or trout parvalbumin in 50 mM Na-carbonate buffer, pH 9.2. After blocking with 3% nonfat dry milk in TBST, the plates were incubated with different concentration of scFv or scFv-AP in TBST containing 1% BSA. Bound scFv-AP was detected directly using p-nitrophenyl phosphate tablet sets (Sigma-Aldrich, Steinheim, Germany). Bound scFv was detected using mouse anti-penta-His IgG1 antibodies (Quiagen, Hilden, Germany), followed by incubation with AP-conjugated rabbit anti-mouse IgG + IgM antibodies (Jackson Immunoresearch, West Grove, PA, USA). Color development was performed was performed as described above.

### Western blotting

Fish protein extracts were separated by 15% SDS-PAGE gel under reducing conditions and electro-transferred onto nitrocellulose membranes. After blocking with 3% nonfat dry milk in TBST, the blots were incubated with 10 μg/ml scFvs or scFv-AP in TBST containing 1% BSA overnight at 4°C. The bound scFvs-AP were vizualised directly by adding the substrate containing 5-bromo-4-chloro-3-indolylphophate (BCIP) and nitroblue tetrazolium (NBT), whereas the bound scFvs were detected using two additional secondary antibodies as described above for the ELISA assay.

For parvalbumin detection by monoclonal and polyclonal antibodies, the blots were incubated with the mouse monoclonal anti-parvalbumin clone Parv-19 antibody (Sigma-Aldrich, Steinheim, Germany) or a rabbit polyclonal anti-Gad m 1 antibody (Tepnel BioSystems Ltd, Deeside, UK). The bound antibodies were detected by AP-conjugated rabbit anti-mouse IgG + IgM or AP-conjugated swine anti-rabbit IgG antibodies (Dako, Glostrup, Denmark), respectively.

### Competitive IgE ELISA

The ability of the Parv-scFv-gco9 and Parv-scFv-goo8 antibodies to inhibit the binding of parvalbumin-specific IgE from fish allergic patients’ sera was tested by ELISA competition experiments. We used sera from three allergic patients who reported clinical symptoms following consumption of various fish species (P1, P2 and P3).

Maxisorp immunoplates were coated with Gad m 1, Cyp c 1 or Onc m 1 as described above. Individual patients’ sera diluted 1:10 containing Parv-scFv-gco9 or Parv-scFv-goo8 at final concentrations of 0, 1, 5 and 20 μg/ml were added to the parvalbumin-coated wells. The plates were washed and binding of IgE was detected with a 1:1000 diluted alkaline phosphatase-conjugated mouse anti-human IgE antibody (BD Pharmingen, San Diego, CA, USA). Color development was performed as described above.

### Analysis of the Gad m 1:scFv-gco9 interaction by NMR spectroscopy

Interaction of [^15^N-^13^C]-labeled rGad m1 and scFv-gco9 was monitored by analysis of ^1^H-^15^N HSQC experiments. The production and the solution structure of the [^15^N-^13^C]-labeled rGad m 1 was previously published by our groups [23].

Gad m 1:scFv-gco9 complex formation was performed in 50 mM sodium phosphate pH 7.5, 150 mM NaCl, with 25% glycerol, at molar ratios Gad m 1:scFv complex of 2:1, and 1:1 and Gad m 1 concentrations of 20 μM, 40 μM and 70 μM. Complex formation was monitored by chemical shift perturbation (CSP), obtained from ^1^H-^15^N HSQC spectra comparison between free and scFv-complexed Gad m 1. The CSP was calculated using the formula *CSP = | ΔδH| + 0*.1*| ΔδN|, where *|ΔδH|* and *Δδ N|* are the CSP of ^1^H and ^15^N nuclei. The molecular dynamics of free Gad m 1 and Gad m 1:scFv-gco9 were monitored by ^15^N backbone relaxation experiments measuring the transversal (R_2_) and longitudinal (R_1_) relaxation rates measured as described in [23].

### *In silico* docking

We performed computational docking simulations of scFv-gco9 and scFv-goo8 versus the fish allergens Gad m 1 and Cyp c 1 to rationalize their interactions at atomic level. Therefore, we constructed a homology model of the scFv antibodies using MOE's antibody modeler tool kit (Molecular Modeling Environment MOE, 2013.08, Chemical Computing Group Inc. 2013, Montreal, Canada) and the included antibody database using default settings and the Amber force field 12:EHT. The modeled antibody structure was then docked to the first conformation of an NMR ensemble of Gad m 1 (PDB: 2MBX) (23) and a crystal structure of Cyp c 1 (PDB: 4CPV) [24].

We used Rosetta's docking protocol (RosettaDock version 3.4) [25] to generate 2000 independent protein-protein docking poses using default settings. Starting structures were generated from random orientations of both binding partners and subjected to a two stage protocol of low resolution exploration using a Monte Carlo protocol and subsequent refinement at an all-atom level. Thereby, smaller conformational adaptions of both binding partners were captured. The ensemble of 2000 docking poses was statistically evaluated in respect to docking scores and predicted binding geometries. All residues with at least one heavy atom with a maximum distance of 5Å to the antibody CDR region were considered as binding part of a epitope.

## Results

### Selection of parvalbumin-specific phages by sequential antigen biopanning

**Fig 1:**
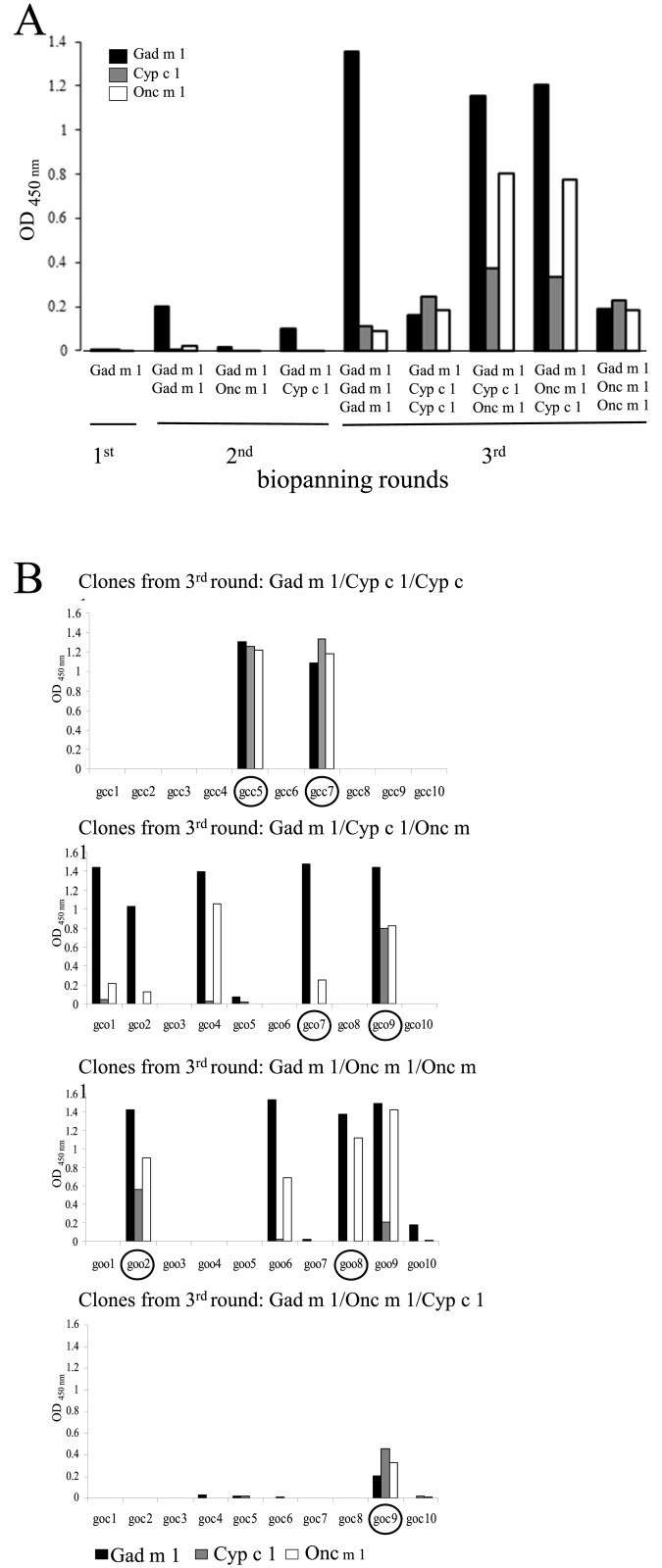
Monitoring the progress of biopanning by polyclonal phage ELISA . (A) The polyclonal phage mixture from each round of biopanning was tested for recognition of Gad m 1, Cyp c 1 and Onc m 1. (B) Monoclonal phage ELISA: Ten single phage clones randomly selected from each third round of biopanning were tested for binding to Gad m 1, Cyp c 1 and Onc m 1.

To isolate cross-reactive scFv antibodies from the ETH-2 phage display library sequential antigen biopanning against purified Gad m 1, Cyp c 1 and Onc m 1 was performed (Table 1). Parvalbumin-specific scFv enrichment was confirmed by polyclonal phage-scFv ELISA using the phage libraries obtained from each biopanning. An increasing response in each round against all three parvalbumins was demonstrated (Fig 1A). In each of the five biopanning series of the third round, strong ELISA signals were observed. The polyclonal phage mixture obtained from the series Gad m1/Cyp c 1/Cyp c 1 and Gad m 1/Onc m 1/Onc m 1 recognized all three parvalbumins equally, whereas phages from the Gad m 1/Cyp c 1/Onc m 1 and Gad m 1/Onc m 1/Cyp c 1 biopannings recognized Gad m 1 and Onc m 1 better than Cyp c 1 parvalbumin.

Subsequntly, ten single phage clones randomly selected from each mixed third round panning were tested for their ability to bind to Gad m 1, Onc m 1 and Cyp c 1 (Fig 1B). Two positive clones from the biopanning series Gad m1/Cyp c 1/Cyp c 1 and one positive clone from Gad m 1/Onc m 1/Cyp c 1 bound all three targets equally well. With exception of two clones (scFv-gco9 and scFv-goo2), positive clones of the series Gad m 1/Cyp c 1/Onc m 1 and Gad m 1/Onc m 1/Onc m 1 showed a preference for Gad m 1 and Onc m 1 (Fig 1B).

**Fig 2:**
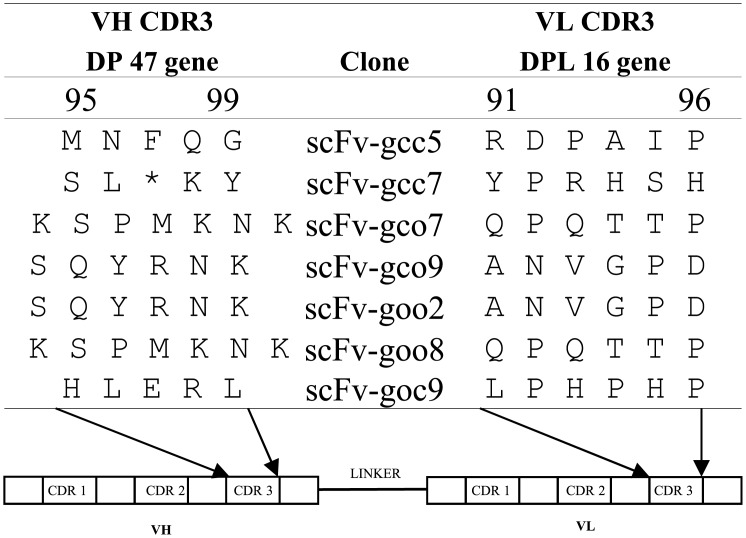
Sequences of the CDR3 regions of the VH and VL chains of the selected scFv-antibodies.

Seven single phage clones (scFv-gcc5, scFv-gcc7, scFv-gco7, scFv-gco9, scFv-goo2, scFv-goo8 and scFv-goc9) with different binding patterns to the three parvalbumins were selected for DNA sequencing. From the two (DPK-22 and DPL-16) human germline genes used to construct the light chain variable region of the ETH-2 library one (DPL-16) was represented. The sequences of the CDR3 regions are shown in Fig 2. Five clones were different in the CDR3 sequences of the variable heavy (V_H_) and light (V_L_) chains. The CDR3 sequence of scFv-gco7was identical to scFv-goo8, and scFv-goo2 was identical to scFv-gco9. A stop codon was noticed in the CDR3 region sequence of clone scFv-gcc7.

In a control experiment to assess the efficiency of the sequential antigen biopanning, biopanning was performed with cod parvalbumin only (Gad m 1/Gad m 1/Gad m 1). In this case, all of 70 clones tested were able to recognize only Gad m 1, and turned out to be identical as determined by DNA sequencing.

### Preparation of soluble scFv antibodies

Based on their ability to bind different parvalbumins and sequence analyses, phage clones scFv-gco9 and scFv-goo8 were selected for the production of soluble scFv antibodies. The phage clone scFv-gco9 bound all three parvalbumins, whereas phage clone scFv-goo8 recognized Gad m 1 and Onc m 1, but not Cyp c 1 (Fig 1B).

**Fig 3:**
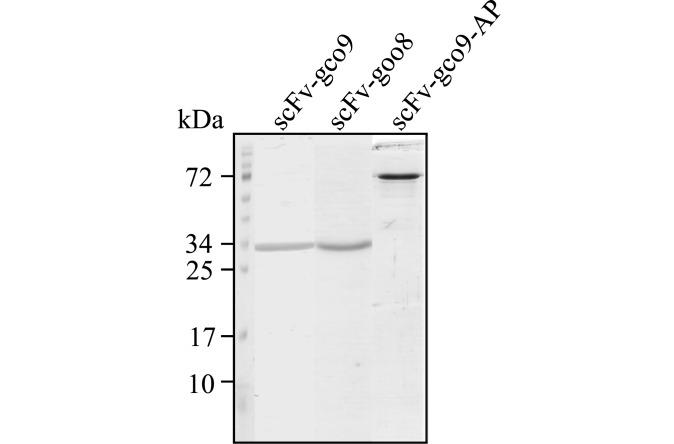
Coomassie brilliant blue-stained SDS-PAGE analyses of the purified scFv antibodies (1 μg/lane).

*E*. *coli* nonsupressor strain HB2151 was inoculated with the selected phage clones for expression as soluble antibodies. Recognition of the amber stop codon between the genes encoding scFv and the M13 pIII coat protein resulted in the production of soluble scFv. The soluble scFv was directed to the periplasma by a pelB leader sequence. The optimized expression of soluble scFv antibodies was performed for 18 h at 30°C. Since scFv antibodies contained a C-terminal 6x his-tag, the soluble antibodies were purified using Ni-NTA. After purification a band of 28 kDa was observed on Coomassie-stained reducing SDS-PAGE with a purity of more than 95% (Fig 3, lanes 1 and 2). We obtained 1 mg of scFv-gco9 and 1.3 mg of scFv-goo8 per litre of bacterial culture.

### Production of the scFv-alkaline phosphatase fusion protein

The phage clone scFv-gco9 was selected for expression as an alkaline phosphatase (AP) fusion protein scFv-gco9-AP. The DNA sequence coding for scFv-gco9 was cloned into the pDAP2/S vector (22) to fuse the scFv to an improved *E*. *coli* alkaline phosphatase (AP/S) and the protein was expressed in *E*.*coli*. The majority of scFv-gco9-AP was secreted in the culture medium, whereas a small amount was retained in the periplasmatic fraction. The culture medium was therefore used by Ni-NTA. In the purified fraction a major band of the expected 75 kDa was present and a yield of approximately 3.4 mg per litre of culture was obtained (Fig 3, lane 3).

### Detection and cross-reactivity of the scFv and bifunctional scFv-AP antibodies

**Fig 4:**
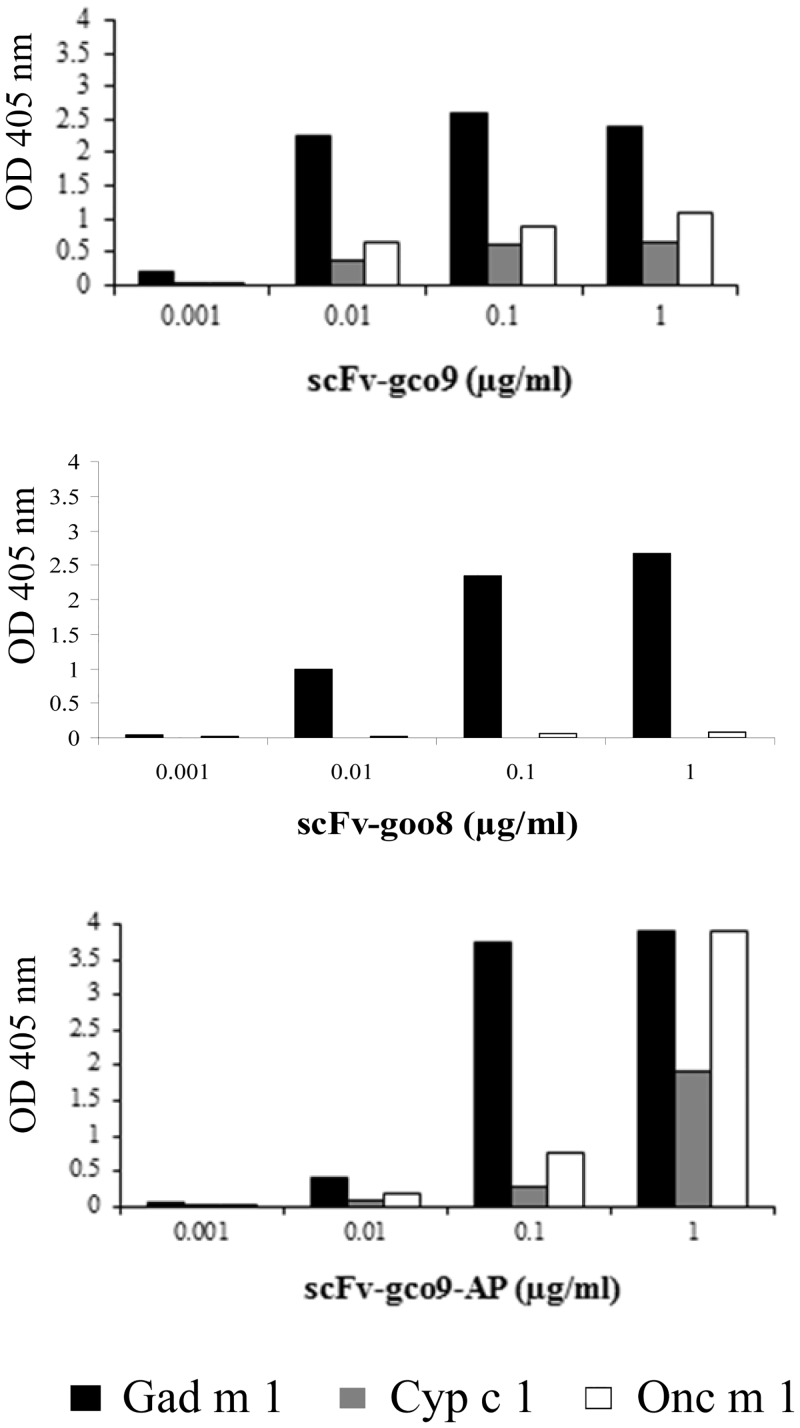
ELISA assay showing the sensitivity of scFv and scFv-AP antibodies. scFv-gco9, scFv-goo8 and scFv-gco9-AP were tested to recognize purified cod, carp and trout parvalbumin. Serial dilutions of purified scFv fragments were used (1 μg/ml–1 ng/ml).

The ability of the soluble scFv antibodies scFv-gco9 and scFv-goo8 and the recombinant alkaline phosphatase fusion antibody scFv-gco9-AP to bind the parvalbumins was analyzed by ELISA (Fig 4). While, the binding to parvalbumins of the scFv-gco9 and scFv-goo8 was detected using anti-penta-His antibodies, following by incubation of AP-conjugated rabbit anti-mouse IgG + IgM antibodies and addition of the enzyme substrate, the binding of the scFv-gco9-AP was simply detected by adding the enzyme substrate. scFv-gco9 and scFv-gco9-AP were able to detect all three parvalbumins of a concentration of 10 ng/ml. scFv-goo8 strongly bound to cod parvalbumin, but in contrast to the result obtained by the monoclonal phage ELISA (Fig 1B) binding to trout parvalbumin was either undetectable or very low.

### Detection of parvalbumins by Western Blot in processed cod, carp and trout

**Fig 5:**
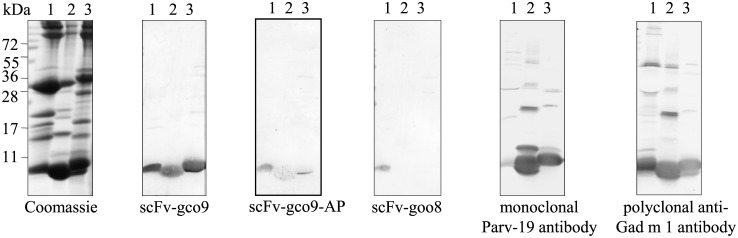
Detection of parvalbumin in processed fish products. Coomassie brilliant blue-stained SDS-PAGE, immunoblotting with scFv-gco9, scFv-goo8, monoclonal- and polyclonal anti-Gad m 1 antibodies: breaded cod sticks (lane 1), fried carp (lane 2), smoked trout (lane 3).

To further characterize the binding specificity of these antibodies, we evaluated the ability of the scFv to recognize parvalbumin in processed fish products such as breaded cod sticks, fried carp and smoked trout in Western blots. The reactivity of the three recombinant antibodies were compared to the reactivity of the commercial mouse monoclonal anti-parvalbumin clone Parv-19 antibody and a commercial rabbit polyclonal anti-Gad m 1 antibody. The protein profiles of the fish products were revealed by Coomassie-stained SDS-PAGE, shown in Fig 5.

All three protein extracts showed a strong band at 12 kDa, corresponding to the size of parvalbumin. scFv-gco9 and scFv-gco9-AP exhibited highly selective binding to parvalbumins in all three fish products. Similar to the ELISA results, scFv-goo8 recognized cod but not carp and trout parvalbumins. The two commercial antibodies recognized equally well the parvalbumins of all three fish products, however they also bound to other different proteins in the extracts (Fig 5).

### Inhibition of IgE-binding to the three parvalbumins by scFv-gco9

In order to find out whether the two recombinant antibodies can compete with binding of IgE from fish-allergic patients’ sera to parvalbumin, a competitive IgE ELISA assay was performed. Three individual patients’ sera were incubated with immobilized parvalbumin together with increasing concentrations of scFv-gco9 or scFv-goo8.

**Fig 6:**
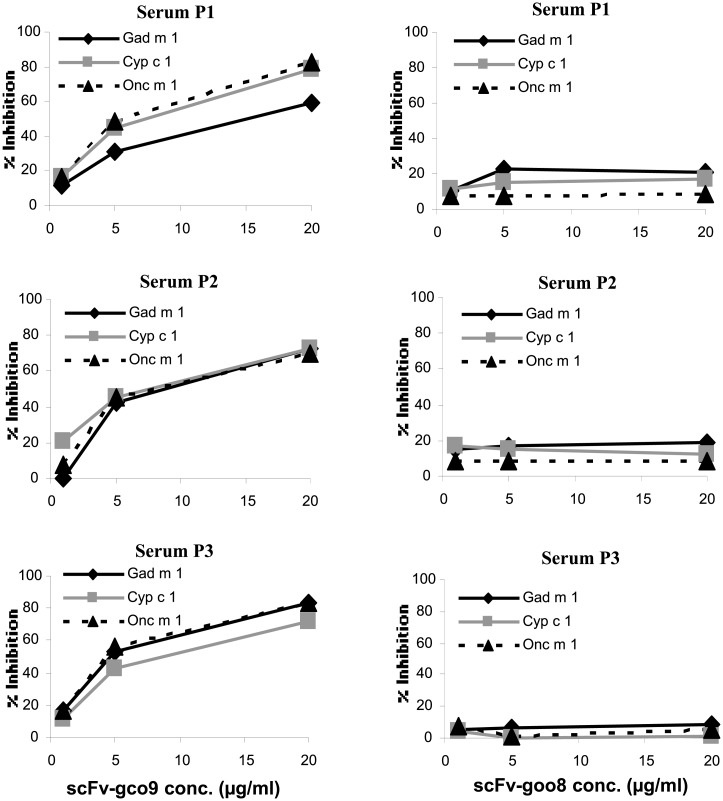
IgE ELISA inhibition assay. The ability of scFv-gco9 and scFv-goo8 to inhibit IgE binding to Gad m 1, Cyp c 1 and Onc m 1 was tested with sera of three fish allergic patients. Sera were preincubated with 1 μg/ml, 5 μg/ml and 20 μg/ml of scFv-gco9 or scFv-goo8 before proceeding with the immunoassay.

We found that scFv-gco9 dose-dependently blocked the binding of IgE to immobilized Gad m 1, Cyp c 1 and Onc m 1 (Fig 6). At a concentration of 5μg/ml of scFv-gco9, binding of IgE to the three parvalbumins was inhibited by approximately 40%, and at a concentration of 20 μg/ml the IgE binding was inhibited to 80% whereas in the case of scFv-goo8, inhibition of IgE binding to Gad m 1 was below 20% with 20 μg/ml competitor concentration for serum No. 1 and 2, and 10% for serum No. 3.

### Mapping of interaction between Gad m 1 and scFv-gco9 by NMR spectroscopy

**Fig 7:**
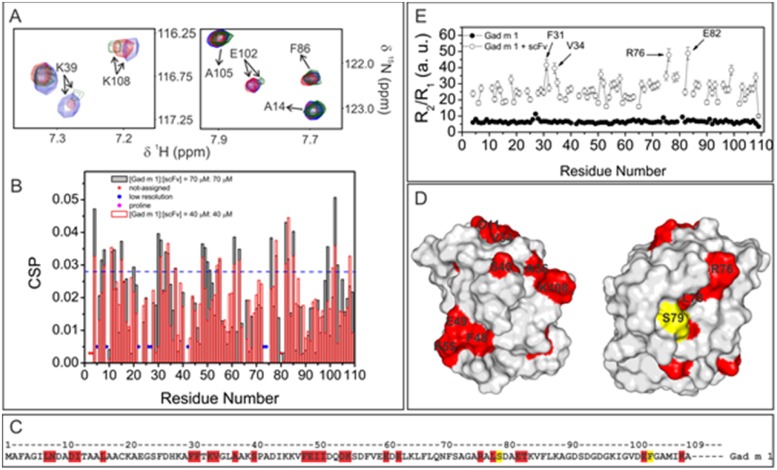
NMR analysis of Gad m 1: scFv-gco9 complex formation. (A) Selected regions of the ^1^H/^15^N HSQC spectra of Gad m 1 in its free form (blue) and in complex with scFv-gco9 at 40 μM or 70 μM complex concentration in red and green, respectively. (B) CSP index as a function of amino acid residue numbers was used to map the residues involved in the interaction in two complex concentrations, 40 μM (red) and 70 μM (gray). (C) Primary sequence of Gad m 1.0202 showing mapped amino acid residues (red) and amino acid residues whose NMR signals disappeared upon complex formation (yellow). (D) Same as in panel C colored in the Gad m 1 NMR solution structure (PDB:2mbx). (E) ^15^N backbone relaxation parameters R_2_/R_1_ of Gad m 1 and the Gad m 1:scFv-gco9 complex as a function of amino acid residue numbers.

Residues involved in the interaction between the scFv and Gad m 1 were identified by the comparison of the ^1^H/^15^N HSQC spectra acquired for the free and bound Gad m 1. Fig 7 shows the Gad m 1 residues that were perturbed in the presence of scFv-gco9, which was dependent on the Gad m 1:scFv-gco9 molar concentration ratio. In panel A, selected regions of ^1^H/^15^N HSQC spectra comparing the shifts of free Gad m 1 and in complex, and in B, the quantitative analysis using CSP calculated from NMR spectra clearly indicated extensive and specific interaction surfaces. Signals from a number of residues, such as Phe-31, Glu-102 and Lys-108, clearly underwent significant shifts on complex formation, whereas others, such as Ala-14, Lys-39 and Ala-105 remained unperturbed. The CSP data showed that from 27 significantly perturbed amino acid residues (shift > 0.028 ppm) five were located at the N-terminus, 16 in the region close to CD loop, five around residue 80 and three at the C-terminus of Gad m 1 (Fig 7, panel C and D). In panel C, the residues with higher CSP values are colored in red in the primary sequence, and in panel D, in the solution structure of Gad m 1 (PDB: 2MBX). The complex formation was also confirmed by substantially different relaxation behavior compared with the relaxation parameters of Gad m 1 (Fig 7, panel E).

**Fig 8:**
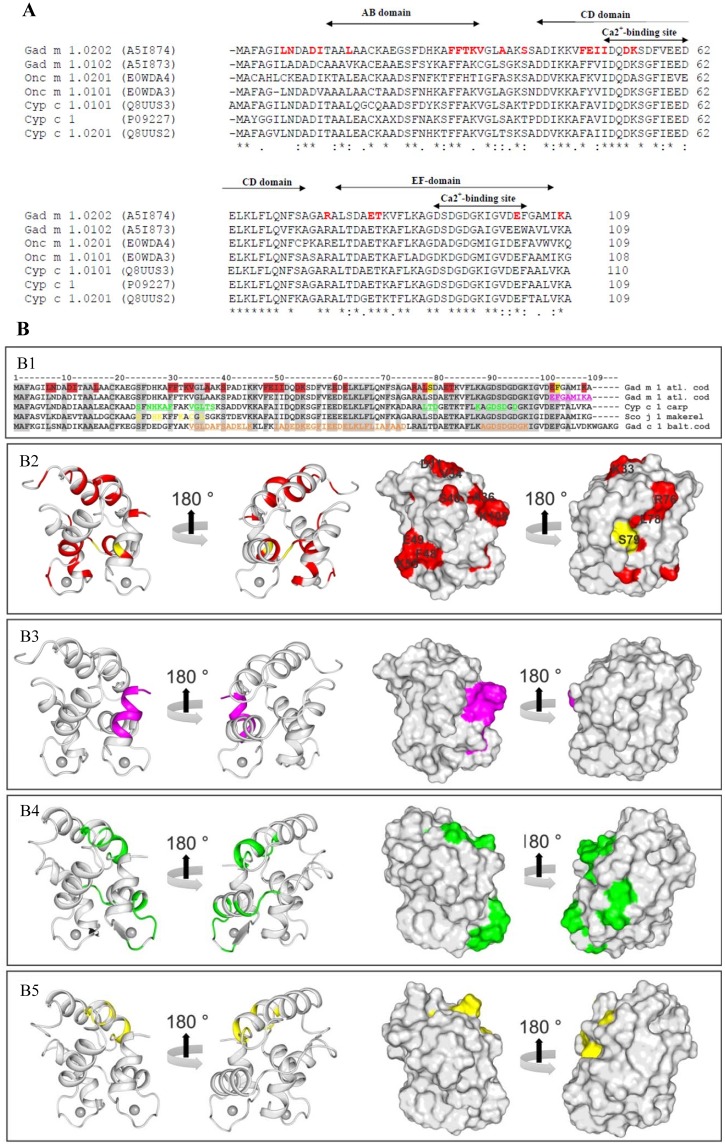
**(A) Protein sequence alignment of Gad m 1, Cyp c 1, Onc m 1 and their isoallergens.** “*” indicates invariant, “:” highly conserved, and “.” weakly conserved residues. EF-hand motifs and calcium-binding sites are indicated by arrows. (B) **Amino acid sequence alignment and mapping of IgE epitopes. (B1)** Gad m 1.0202 (Acc. number: A5I874), Cyp c 1 (Acc. number: P02618), and Sco j 1 (Acc. number: P59747), Gad c 1 (Acc. number: P02622). Amino acid residues involved in Gad m1.0202-scFv-complex formation and known IgE-binding regions of the parvalbumins are colored in red, pink, green, yellow and orange, respectively. The same residues were mapped onto the protein structures: **(B2)** and **(B3)** Gad m 1.0202 (PDB ID: 2mbx); **(B4)** Cyp c 1 (PDB ID: 4cpv), and **(B5)** Sco j 1. Since the three-dimensional structure of Sco j 1 was not available, a structure model was built using Swiss-Model server and 2mbx as a template.

Because scFv-gco9 cross-reacted with Cyp c 1 from carp and Onc m 1 from trout, scFv-gco9 is expected to bind to conserved residues among those three parvalbumins. Fig 8A shows that many residues involved in the interaction of Gad m 1 and scFv-gco9 are conserved in primary sequences of these parvalbumins (Fig 8A).

To validate the ability of scFv-gco9 to inhibit IgE-binding, we compared our NMR results to previously reported IgE-binding sites from Gad m 1 [26], Gad c 1 [27], Cyp c 1 [14, 28], and Sco j 1 [29]. Fig 8B panel B1 shows a sequence alignment of Gad c 1, Gad m 1, Cyp c 1, and Sco j 1 with the identified epitopes. Residues around positions 30 to 40 matched IgE epitopes mapped for the three allergens. IgE-binding to residues around position 50 to 60 were only observed for Gad m 1 and Gad c 1, while residues around 80 were mapped on Cyp c 1. Furthermore, the accessibility of the mapped residues was evaluated (Fig 8B panels B2, B3, B4 and B5). The Gad m 1 conformational epitope including residues 7, 8, 11, 12, 15, 29–33, 36, and 108 mapped for scFv-gco9 was also mapped as IgE-binding site of Gad m 1, Gad c 1 and Cyp c 1 using overlapping peptides. The IgE epitope mapped on Sco j 1 was less similar to the regions probed in Gad m 1.

### Computational epitope mapping of Gad m1 and Cyp c 1

**Fig 9:**
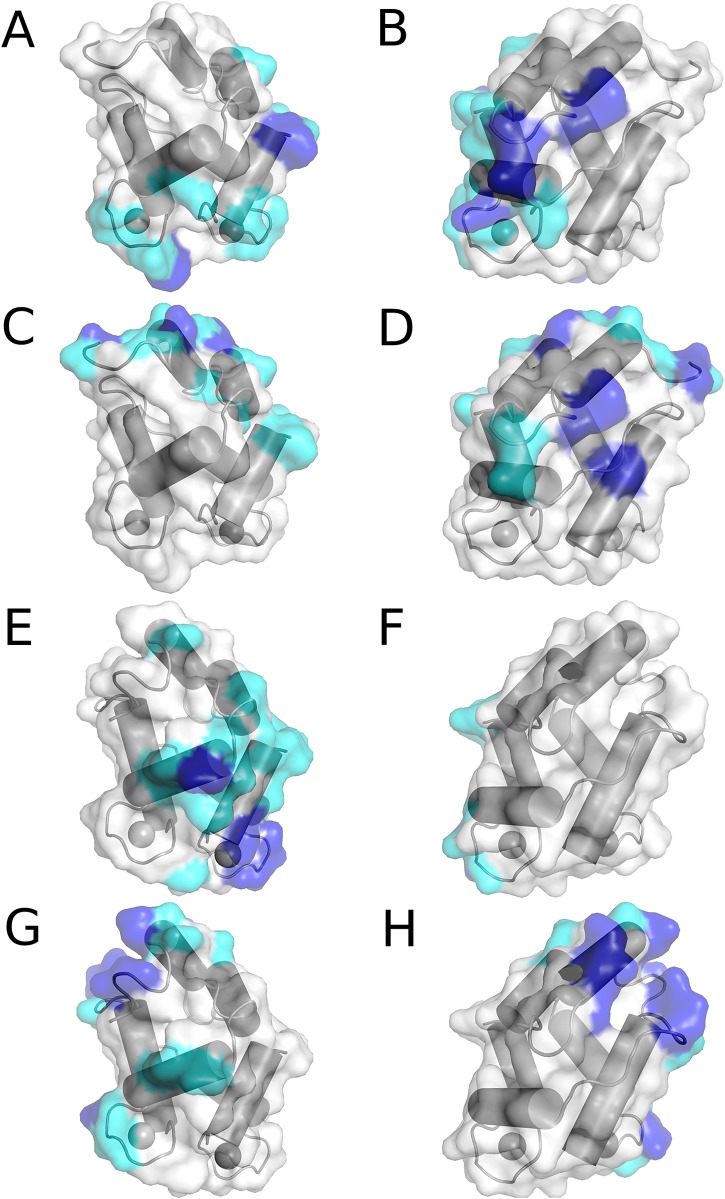
Interacting regions from *in silico* allergen-antibody docking. Residues strongly involved in molecular interactions are highlighted on a color ramp from white (no interactions) via cyan to blue (many interactions). **(A, B)** Predicted strong interactions between the Gad m 1 and scFv-gco9 allergen in two orientations. **(C, D)** Similar parts of Gad m 1 are also targeted by scFv-goo8. These regions are also involved in the scFv-gco9 binding to Cyp c 1 **(E, F)**. Weaker signals and a broader distribution of involved residues observed for Cyp c 1 as compared to Gad m 1. **(G, H)**. In silico docking of scFv-goo8 to Cyp c 1 reveals a change in the binding epitope. The major epitope at the C-terminal is less targeted. Instead, a region close to the N-terminal is accessible to antibody binding.

Based on 2000 predicted protein-protein dockings, we aimed to rationalize cross-reactivity of the scFv-gco9 as well as selectivity of scFv-goo8. We found a broad and overlapping distribution of docking scores between both systems and, therefore, focused our analysis on geometrical parameters. We mapped residues frequently involved in scFv binding amongst the 2000 predicted complexes to the protein structures (Fig 9). In agreement with the CSP data, we observed strong binding signals for the region around residue 60 and the C-terminal residues of Gad m 1 for both antibodies. Single residues identified around residue 80 are also in agreement with CSP data. Additionally, we found many interactions mediated by residues 20–30 which has not been observed in the NMR studies of Gad m 1.

For Cyp c 1, we found a broader distribution of binding poses and thus less pronounced signals for single residues which points towards a weaker binding of scFv-gco9 to this protein. Nevertheless, we found many residues at the C-terminus of Cyp c 1 to be involved in scFv-gco9 recognition, thus overlapping with results for Gad m 1. The strongest signal was observed for a region around residue 80 which is in agreement with experimental data for the system. Furthermore, we found many residues around residue 40 to be involved in another interaction site of the scFv-gco9 in Cyp c 1. In contrast to published experimental data, residues 20–30 are mostly not predicted as binding epitopes. In contrast to binding of scFv-gco9 binding, we observe a change in binding epitopes for scFv-goo8. Here, the prominent epitope near the C-terminal is less frequently targeted during docking. Instead, residues 3–10 are predicted as a major region for binding which has not been observed for the other antibody-antigen binding simulations.

## Discussion

Fish parvalbumins are the main cause of often severe IgE-mediated symptoms in 0.1–0.5% of the general population [1–3]. Here we describe an approach for the selection of monoclonal parvalbumin-specific scFv antibodies by phage display technology. We show the successful use of such recombinant antibodies for the detection of allergen-inducing fish parvalbumins in processed food products as well as their applicability for the characterization of IgE-binding epitopes.

Commercially available polyclonal and monoclonal parvalbumin-specific antibodies are considered as useful tools for detecting parvalbumins in fish extracts and fish-containing products [30, 31]. Polyclonal antibodies directed against parvalbumins from cod or barramundi appeared to be suitable for the detection of parvalbumins [16, 17, 32, 33]

The anti-barramundi parvalbumin proved to be the most cross-reactive antibody, detecting 87.5% of 40 fish species analysed [34]. In the case of monoclonal antibodies such as the anti-frog (MAb PARV-19) and the anti-catfish (MAb 3E1) parvalbumin antibodies, the observed cross-reactvity was low [32–35]. In case of the MAb PARV-19, non-allergenic parvalbumins from rabbit and rat were also recognized [33].

Recombinant scFv antibodies selected by phage display offer a rapid and economical alternative to the laborious production of monoclonal antibodies and the batch to batch variations of polyclonal antibodies. We found that biopanning of the human antibody phage library ETH-2 against cod parvalbumin resulted in the selection of clones which were exclusively specific for Gad m 1. Consequently, we hypothesized that by changing the targets during biopanning, the selected phages would display scFv against conserved epitopes shared among all antigens. For this purpose we used parvalbumins from three commercially important and frequently consumed fish species [5]belonging to three phylogenetically different orders, Gadiformes (cod), Cypriniformes (carp) and Salmoniformes (rainbow trout). The results showed a progressive enrichment of different scFv clones binding epitopes conserved among all three parvalbumins.

Following the successful expression and purification of the two selected phage clones named scFv-gco9 recognizing all three parvalbumns and scFv-goo8 recognizing predominantly cod parvalbumin. The antibody scFv-gco9 strongly recognized natural Gad m 1, Cyp c 1 and Onc m 1 at a concentration of 10 ng/ml, while scFv- goo8 recognized exclusively Gad m 1 suggesting that the two clones recognize different epitopes. A higher binding to Onc m 1 of the phage clone goo8 compared with soluble scFv-goo8 antibody indicates better stability and folding of this scFvs expressed as a fusion with the phage pIII coat protein.

In order to obtain efficient a detecting reagent, the coding sequence for scFv-gco9 was subcloned into the expression vector pDAP2/S [22] and produced as an alkaline phosphatase fusion antibody. A combination of the alkaline phosphatase enzymatic activity and the antigen-binding ability of the recombinant antibody made a single-step detection of cod, carp and trout parvalbumins possible as there was no need for a secondary antibody. The results clearly showed that the fusion protein scFv-gco9-AP retained both enzyme activity and antigen binding activity.

Another aim was to determine the ability of the selected antibodies to inhibit binding of IgE from sera of fish allergic patients to cod, carp and trout parvalbumins. In a competitive ELISA, the scFv-gco9 antibody, in contrast to scFv-goo8, was able to inhibit the binding of IgE from fish allergic patients’ sera to all three parvalbumins by up to 80%. The high inhibitory effect of the scFv-gco9 on the binding of parvalbumin-specific IgE suggested that inhibition was either caused by direct competition of the scFv and IgE for the same epitope, by partial overlap of the scFv and IgE binding site, or by perturbation of IgE epitopes by conformational changes induced by scFv binding.

Previous studies on parvalbumins from Baltic cod (Gad c 1) [27], Atlantic cod (Gad m 1) [26], carp (Cyp c 1) [14, 28], and mackerel (Sco j 1) [29] using various techniques including tryptic digests, peptide phage display library, site directed mutagenesis, and overlapping peptides to map IgE binding sites, demonstrated the presence of different IgE epitopes of both linear and conformational types. This may be due to the varying techniques utilized to identify these epitopes as well as to the polyclonal nature of IgE antibodies from different patients. An earlier study by Elaysed et al. on Gad c 1 [27] as well as a recent study by Perez-Gordo et al. on Sal s 1 from Atlantic salmon) [36] showed that a peptide corresponding to amino acids 28–45 (the axis joining the AB and CD motifs) contained IgE binding sites. Again using overlapping peptides, another study identified a dominant IgE binding peptide located at the C-terminus (amino acids 95–109) of Gad m 1 [26]. Swoboda et al. showed a general strategy for generation of non-IgE binding parvalbumins by introducing 4 point mutations into the two calcium-binding regions of different parvalbumins [15]. Exchange of four conserved aspartic acids by alanins resulted in the loss of IgE-binding also of distantly related parvalbumins, indicating the general conformational nature of parvalbumin IgE epitopes.

As single chain antibodies are much smaller than immunoglobulins they can be used for epitope mapping by NMR spectroscopy. The NMR analysis of Gad m 1:scFv-gco9 complexes revealed participation of amino acids conserved among the three distantly related parvalbumins thus confirming the molecular basis of the observed cross-reactivity of the scFv. Furthermore, regions of Gad m 1 comprising amino acid residues exhibiting significant CSPs in the Gad m 1:scFv-gco9 complex have been previously identified as IgE-binding peptides [26, 27, 36]. Although the identified amino acid residues close to the N-terminus, the axis joining AB and CD domain and close to the C-terminus are far distant on the linear sequence, they are in close proximity in the 3-dimensional structure indicating a possible conformational nature of the scFv epitope. In silico docking data is largely in agreement with the presented CSP data and published epitope mappings for Gad m 1 and Cyp c 1. For both allergens we found the C-terminal region to be a key region for antigen-antibody recognition. This region appears to be less prominent in scFv-goo8—Cyp c 1 interactions, thus providing a rationale for the antibody's selectivity for Gad m 1. However, one limitation of the approach is the lack of direct experimental data to define the amino acids residues directly involved in the Gad m 1-scFv interface. The Gad m 1:scFv-gco9 interactions involving side chains should therefore be viewed as reasonable possibilities, rather than confirmed interactions. However, the data provide a good basis for the assessment of possibly key interactions by further experimental work, such as site directed mutagenesis.

In conclusion, we present a simple approach for selection of cross-reactive scFv antibodies by phage display technology by sequentially exchanging parvalbumins from distantly related fish species during biopanning and its application to allergen detection and analysis of IgE epitopes.

## Abbreviations

AP: alkaline phosphatase
CSP: chemical shift perturbation
ELISA: enzyme linked immunosorbent assay
IPTG: isopropyl β-D-1-thiogalactopyranoside
scFv: single chain variable fragment

## References

[1] Nwaru BI, Hickstein L, Panesar SS, Roberts G, Muraro A, Sheikh A. Prevalence of common food allergies in Europe: a systematic review and meta-analysis. Allergy. 2014;69(8):992–1007. 10.1111/all.12423 24816523

[2] Soller L, Ben-Shoshan M, Harrington DW, Fragapane J, Joseph L, St Pierre Y, et al Overall prevalence of self-reported food allergy in Canada. J Allergy Clin Immunol. 2012;130(4):986–8. 10.1016/j.jaci.2012.06.029 22867693

[3] McGowan EC, Keet CA. Prevalence of self-reported food allergy in the National Health and Nutrition Examination Survey (NHANES) 2007–2010. J Allergy Clin Immunol. 2013;132(5):1216–9. 10.1016/j.jaci.2013.07.018 23992749 PMC3822433

[4] Sharp MF, Lopata AL. Fish allergy: in review. Clin Rev Allergy Immunol. 2014;46(3):258–71. 10.1007/s12016-013-8363-1 23440653

[5] Failler P. Future prospects for fish and fishery products Fish consumption in the European Union in 2015 and 2030. FAO Fisheries Circular. Rome: FAO; 2007.

[6] Nwaru BI, Hickstein L, Panesar SS, Muraro A, Werfel T, Cardona V, et al The epidemiology of food allergy in Europe: a systematic review and meta-analysis. Allergy. 2014;69(1):62–75. 10.1111/all.12305 24205824

[7] Bugajska-Schretter A, Elfman L, Fuchs T, Kapiotis S, Rumpold H, Valenta R, et al Parvalbumin, a cross-reactive fish allergen, contains IgE-binding epitopes sensitive to periodate treatment and Ca2+ depletion. J Allergy Clin Immunol. 1998;101):67–74. 9449503 10.1016/S0091-6749(98)70195-2

[8] Pascual C, Martin Esteban M, Crespo JF. Fish allergy: evaluation of the importance of cross-reactivity. J Pediatr. 1992;121:S29–34. 1447631 10.1016/s0022-3476(05)81403-9

[9] Swoboda I, Bugajska-Schretter A, Verdino P, Keller W, Sperr WR, Valent P, et al Recombinant carp parvalbumin, the major cross-reactive fish allergen: a tool for diagnosis and therapy of fish allergy. J Immunol. 2002;168(9):4576–84. 11971005 10.4049/jimmunol.168.9.4576

[10] Kretsinger RH, Nockolds CE. Carp muscle calcium-binding protein. II. Structure determination and general description. J Biol Chem. 1973;248(9):3313–26. 4700463

[11] de Martino M, Novembre E, Galli L, de Marco A, Botarelli P, Marano E, et al Allergy to different fish species in cod-allergic children: in vivo and in vitro studies. J Allergy Clin Immunol. 1990;86:909–14. 2262645 10.1016/s0091-6749(05)80154-x

[12] Van Do T, Elsayed S, Florvaag E, Hordvik I, Endresen C. Allergy to fish parvalbumins: studies on the cross-reactivity of allergens from 9 commonly consumed fish. J Allergy Clin Immunol. 2005;116(6):1314–20. 16337465 10.1016/j.jaci.2005.07.033

[13] Griesmeier U, Vazquez-Cortes S, Bublin M, Radauer C, Ma Y, Briza P, et al Expression levels of parvalbumins determine allergenicity of fish species. Allergy. 2010;65(2):191–8. 10.1111/j.1398-9995.2009.02162.x 19796207

[14] Untersmayr E, Szalai K, Riemer AB, Hemmer W, Swoboda I, Hantusch B, et al Mimotopes identify conformational epitopes on parvalbumin, the major fish allergen. Mol Immunol. 2006;43(9):1454–61. 16150491 10.1016/j.molimm.2005.07.038

[15] Swoboda I, Balic N, Klug C, Focke M, Weber M, Spitzauer S, et al A general strategy for the generation of hypoallergenic molecules for the immunotherapy of fish allergy. J Allergy Clin Immunol. 2013;132(4):979–81. 10.1016/j.jaci.2013.04.027 23763969

[16] Jenkins JA, Breiteneder H, Mills EN. Evolutionary distance from human homologs reflects allergenicity of animal food proteins. J Allergy Clin Immunol. 2007;120(6):1399–405. 17935767 10.1016/j.jaci.2007.08.019

[17] Lapteva YS, Uversky VN, Permyakov SE. Sequence microheterogeneity of parvalbumin, the major fish allergen. Biochimica Et Biophysica Acta-Proteins and Proteomics. 2013;1834(8):1607–14. 10.1016/j.bbapap.2013.04.025 23632315

[18] Ma Y, Griesmeier U, Susani M, Radauer C, Briza P, Erler A, et al Comparison of natural and recombinant forms of the major fish allergen parvalbumin from cod and carp. Mol Nutr Food Res. 2008;52 Suppl 2:S196–207. 10.1002/mnfr.200700284 18504705

[19] Pini A, Viti F, Santucci A, Carnemolla B, Zardi L, Neri P, et al Design and use of a phage display library—Human antibodies with subnanomolar affinity against a marker of angiogenesis eluted from a two-dimensional gel. Biol Chem.1998;273(34):21769–76. 10.1074/jbc.273.34.21769 9705314

[20] Viti F, Nilsson F, Demartis S, Huber A, Neri D. Design and use of phage display libraries for the selection of antibodies and enzymes. Applications of Chimeric Genes and Hybrid Proteins, 2000;326:480–505. 10.1016/s0076-6879(00)26071-0 11036659

[21] Gruber P, Gadermaier G, Bauer R, Weiss R, Wagner S, Leonard R, et al Role of the polypeptide backbone and post-translational modifications in cross-reactivity of Art v 1, the major mugwort pollen allergen. Biol Chem. 2009;390(5–6):445–51. 10.1515/BC.2009.063 19361284

[22] Kerschbaumer RJ, Hirschl S, Kaufmann A, Ibl M, Koenig R, Himmler G. Single-chain Fv fusion proteins suitable as coating and detecting reagents in a double antibody sandwich enzyme-linked immunosorbent assay. Anal Biochem. 1997;249(2):219–27. 9212874 10.1006/abio.1997.2171

[23] Moraes AH, Ackerbauer D, Kostadinova M, Bublin M, de Oliveira GA, Ferreira F, et al Solution and high-pressure NMR studies of the structure, dynamics, and stability of the cross-reactive allergenic cod parvalbumin Gad m 1. Proteins. 2014;82(11):3032–42. 10.1002/prot.24664 25116395

[24] Kumar VD, Lee L, Edwards BFP. Refined Crystal-Structure of Calcium-Liganded Carp Parvalbumin 4.25 at 1.5-a Resolution. Biochemistry. 1990;29(6):1404–12. 2334704 10.1021/bi00458a010

[25] Chaudhury S, Berrondo M, Weitzner BD, Muthu P, Bergman H, Gray JJ. Benchmarking and Analysis of Protein Docking Performance in Rosetta v3.2. Plos One. 2011;6(8). 10.1371/journal.pone.0022477 PMC3149062 21829626

[26] Perez-Gordo M, Pastor-Vargas C, Lin J, Bardina L, Cases B, Ibanez MD, et al Epitope mapping of the major allergen from Atlantic cod in Spanish population reveals different IgE-binding patterns. Mol Nutr Food Res. 2013;57(7):1283–90. 10.1002/mnfr.201200332 23554100

[27] Elsayed S, Apold J. Immunochemical analysis of cod fish allergen M: locations of the immunoglobulin binding sites as demonstrated by the native and synthetic peptides. Allergy. 1983;38(7):449–59. 6356964 10.1111/j.1398-9995.1983.tb02353.x

[28] Swoboda I, Bugajska-Schretter A, Linhart B, Verdino P, Keller W, Schulmeister U, et al A recombinant hypoallergenic parvalbumin mutant for immunotherapy of IgE-mediated fish allergy. J Immunol. 2007;178(10):6290–6. 17475857 10.4049/jimmunol.178.10.6290

[29] Yoshida S, Ichimura A, Shiomi K. Elucidation of a major IgE epitope of Pacific mackerel parvalbumin. Food Chemistry. 2008;111(4):857–61.

[30] Faeste CK, Plassen C. Quantitative sandwich ELISA for the determination of fish in foods. J Immunol Methods. 2008;329(1–2):45–55. 17980385 10.1016/j.jim.2007.09.007

[31] Lopata AL, Jeebhay MF, Reese G, Fernandes J, Swoboda I, Robins TG, et al Detection of fish antigens aerosolized during fish processing using newly developed immunoassays. Int Arch Allergy Immunol. 2005;138(1):21–8. 16088209 10.1159/000087354

[32] Lee PW, Nordlee JA, Koppelman SJ, Baumert JL, Taylor SL. Evaluation and comparison of the species-specificity of 3 anti-parvalbumin IgG antibodies. J Agric Food Chem. 2011;59(23):12309–16. 10.1021/jf203277z 21999209

[33] Sharp MF, Stephen JN, Kraft L, Weiss T, Kamath SD, Lopata AL. Immunological cross-reactivity between four distant parvalbumins-Impact on allergen detection and diagnostics. Mol Immunol. 2015;63(2):437–48. 10.1016/j.molimm.2014.09.019 25451973

[34] Chen L, Hefle SL, Taylor SL, Swoboda I, Goodman RE. Detecting fish parvalbumin with commercial mouse monoclonal anti-frog parvalbumin IgG. J Agric Food Chem. 2006;54(15):5577–82. 16848548 10.1021/jf060291g

[35] Gajewski KG, Hsieh YH. Monoclonal antibody specific to a major fish allergen: parvalbumin. J Food Prot. 2009;72(4):818–25. 19435232 10.4315/0362-028x-72.4.818

[36] Perez-Gordo M, Lin J, Bardina L, Pastor-Vargas C, Cases B, Vivanco F, et al Epitope Mapping of Atlantic Salmon Major Allergen by Peptide Microarray Immunoassay. Int Arch Allergy Immunol. 2012;157(1):31–40. 10.1159/000324677 21894026

